# Catastrophic Health Expenditure in Iran: A Review Article

**Published:** 2018-02

**Authors:** Aidin ARYANKHESAL, Manal ETEMADI, Mohammad MOHSENI, Saber AZAMI-AGHDASH, Majid NAKHAEI

**Affiliations:** 1. Dept. of Health Services Management, School of Health Management and Information Sciences, Iran University of Medical Sciences, Tehran, Iran; 2. Health Management and Economics Research Center, Iran University of Medical Sciences, Tehran, Iran; 3. Iranian Center of Excellence in Health Management, School of Management and Medical Informatics, Tabriz University of Medical Sciences, Tabriz, Iran; 4. Health Services Management Research Center, Institute for Futures Studies in Health, Kerman University of Medical Sciences, Kerman, Iran

**Keywords:** Catastrophic payments, Health economics, Financially vulnerable people, Iran

## Abstract

**Background:**

One of the main challenges of healthcare systems is to protect people from consequences of health expenditures. Such expenditures may lead to catastrophic financial loss in families so that many people deny demanding necessary healthcare services, which results in harms to their health status. The aim of this systematic review was to investigate the catastrophic health expenditures trend and its related factors in Iran.

**Methods:**

This systematic review and meta-analysis was conducted on studies conducted between 1984 and 2014. Data were collected through searching electronic databases and searching engines of PubMed, Scopus, EconLit, Google Scholar, Science Direct, MagIran, and Scientific Information Database (SID). The random effects were used with 95% confidence interval for the meta-analysis.

**Results:**

Out of 561 initially retrieved articles, finally 42 were included in the final analysis. The studies were conducted between 1984 and 2014. The overall proportion of exposure to catastrophic health expenditure in Iran was 7.5% (95% CI, 6.2 – 9.1). In the urban and rural areas, the proportion was 2.3% (95% CI, 1.8 – 2.9) and 3.4% (95% CI, 2.8 – 4.1) respectively. The overall proportion of exposure to the catastrophic health expenditure in hospitals was 35.9% (95% CI, 23.5 – 54.3).

**Conclusion:**

The catastrophic expenditures proportion of healthcare is relatively high in Iran and the government is expected to adopt effective measures in this regard, especially for the inpatient care. There are needs for special supporting policies for the financial protection of specific patients, the poor and villagers.

## Introduction

Health is one of the main pillars of sustainable development (1). Provision of optimal health addressing the public expectations along with financial protection is one of the main responsibilities of health systems (2).

Equitable health financing, protection of people against consequences of health expenditures and the assurance of equity in the utilization of health services are the main challenges of health systems. Lack of comprehensive and universal health coverage usually damages households with lower capacity to afford healthcare services by irreversible health harms (3). In addition, selection among funding and financing methods is one of the key policies for health policymakers in all nations. Direct Out of Pocket Payment (OPP) model usually has been detected as one of the causing factors of catastrophic health expenditures (4). Instead, prepayment is known as key indicators of progress toward universal coverage (5).

According to the WHO’s definition, catastrophic health expenditure occurs when households’ payments on health reach to at least 40% the family’s nonfood expenditures (6). Indeed, the WHO also defines the monetary value of non-food expenditure as a proxy for payment affordability for health costs, and these counts as the denominator for assessing catastrophic expenditures. The indicator for the catastrophe estimation is the level of health expenditure from the total costs and this proportion is compared with the threshold level to determine the percentage of households who incur health catastrophic expenditures (7). The incidence of catastrophic payments can be estimated from the proportion of households with health care costs as a share of nonfood expenditure more than the determined threshold (8).

In 2013, about 10% of total gross domestic products of countries were spent on health of which about one-fifth (18.6%) were financed through OPP by individuals (9). The relationship between the household's payment proportion and incidence of catastrophic or impoverishing expenditures is usually examined and studied by healthcare policymakers (10). The cost-to-income proportion may be high for some families and leads catastrophic financial losses and poverty for them (11). Due to such negative consequences and lack of affordability of healthcare expenditures, many people may deny demanding healthcare services, especially elective ones (12).

Health expenditures in low-income countries are mainly financed through OPP and with few pre-payment mechanisms such as health insurance premium (13, 14). Dependence on OPP payments can impose catastrophic or impoverishing expenditures on households (13). The proportion of households facing catastrophic expenditures varies in different countries, ranging from 1% to 15% (15–17). In separate studies conducted in Brazil and Burkina Faso, percentages of whom facing catastrophic health expenditures were 12% and 15%, respectively (18, 19). In addition, the proportion of exposure to catastrophic expenditures among the rural and urban households in Iran varied from 0.5% to 14.3% and from 0.48% to 13.27%, respectively (20).

Exposure to catastrophic expenditure is reported controversially in Iran, ranging from 1.56 to 72.5 (21, 22). Several factors influence such differences considered in order to achieve an accurate and generalizable estimate for catastrophic health expenditure in Iran. The setting of studies is one of the factors that can explain the estimated differences. For example, in a study conducted at hospitals, the exposure was estimated 72.5, while other study reported the exposure at the community 6.77% (23).

In addition, studies that conducted in particular geographic areas with special focus on certain cities or provinces, with different socio-economic status, can increase the discrepancy of the estimates. In addition, the difference in the number of samples taken in various studies could also cause variations in the estimation of catastrophic expenditures exposure. For example, a study (24) was conducted with 402 urban and rural households in Qazvin Province and exposure to catastrophic expenditures was estimated 9.7, while in another study (25) in Qazvin with a sample of 100 households, the ratio was estimated as 24.

Monitoring and detecting the incidence of catastrophic expenditures and factors affecting them can help policymakers in the health system to adopt proper mechanisms to prevent and resolve this issue. The percentage of catastrophic health expenditure is reported differently through the conducted studies and the differences among findings of the studies we aimed to give a clear estimation of catastrophic health expenditures trend and the related factors in the country for evidence-informed policymaking on financial protection, through a systematic review and meta-analysis.

## Methods

This systematic review and meta-analysis was conducted on studies conducted between 1984 and 2014. The main aim of this review was to estimate the national proportion of exposure to catastrophic expenditures in Iran and its tolerance based on some parameters such as type of services, setting, and type of data sources.

A search strategy was developed assisted by an experienced librarian. Data were collected using a search strategy of words “catastrophic”, “catastrophic health expenditure”, “fair financial contribution”, “cost”, “Iran”, “expenditure”, “poverty”, “inequality”, “payment” and their Persian synonyms across electronic databases and searching engines of PubMed, Scopus, EconLit, Google Scholar, Science Direct, MagIran, and Scientific Information Database (SID). The reference lists of the retrieved articles, certain relevant journals, and websites in the field of health economics were hand-searched.

The inclusion criteria were cross-sectional studies that indicated catastrophic payment proportion based on national, provincial or any setting of Iran, and the languages of English or Persian. No time limitation was considered for the published studies. Letters to the editors, presentations at conferences, and case reports were excluded.

To assess the quality, two authors evaluated the articles according to Newcastle–Ottawa Scale (NOS) (26). In the first phase, articles with non-relevant titles on the subject of the study were excluded. In the second phase, the abstract and the full text of articles were reviewed. Computer software for reference management (Endnote X6) was used for organizing and recognizing the duplications. The collected data were summarized in previously designed extraction tables. To estimate the proportion of catastrophic expenditure a quantitative meta-analysis method was done by computer software (CMA: 2-Comprehensive Meta-analysis).

Forest plot with a 95% confidence interval was used to estimate the overall proportion of catastrophic expenditure. Random effect was used to perform meta-analyses. I^2^ was used to evaluate heterogeneity of studies (27).

## Results

**Fig. 1:**
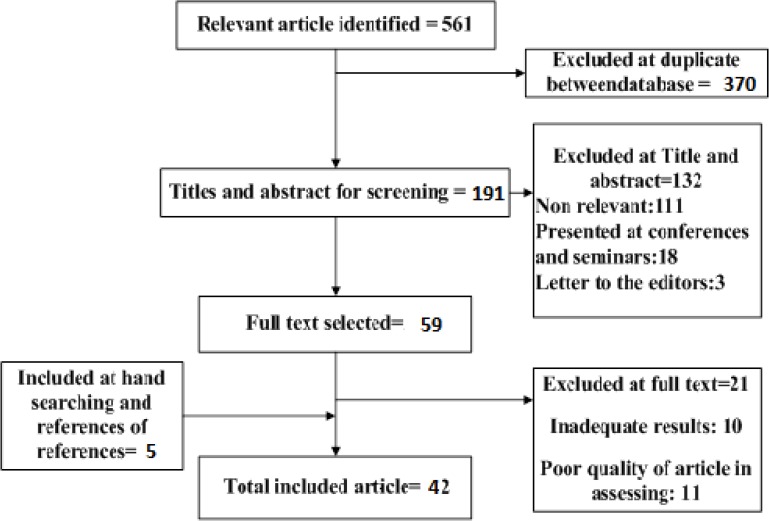
Bibliographical searches and inclusion process

In current study, of the 561 articles screened, totally 39 eligible studies met the inclusion criteria were entered into Meta-Analysis (whit 1262651 cases) and 42 into the systematic (11, 21–25, 28–63). (Fig. 1).

**Fig. 2:**
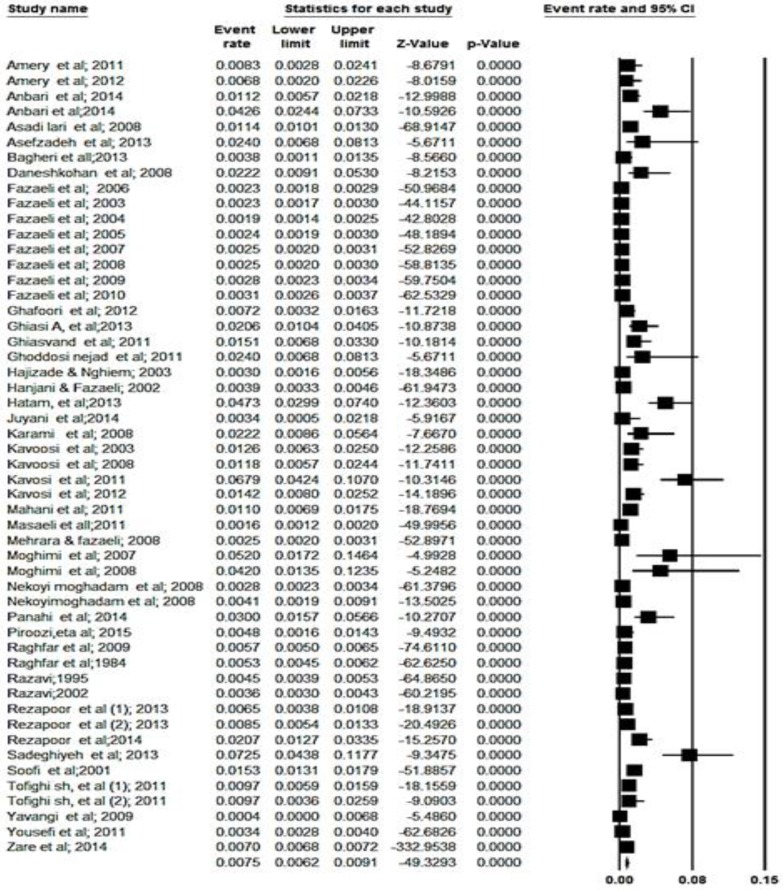
The overall percent of households exposed to catastrophic health expenditures in Iran

**Fig. 3:**
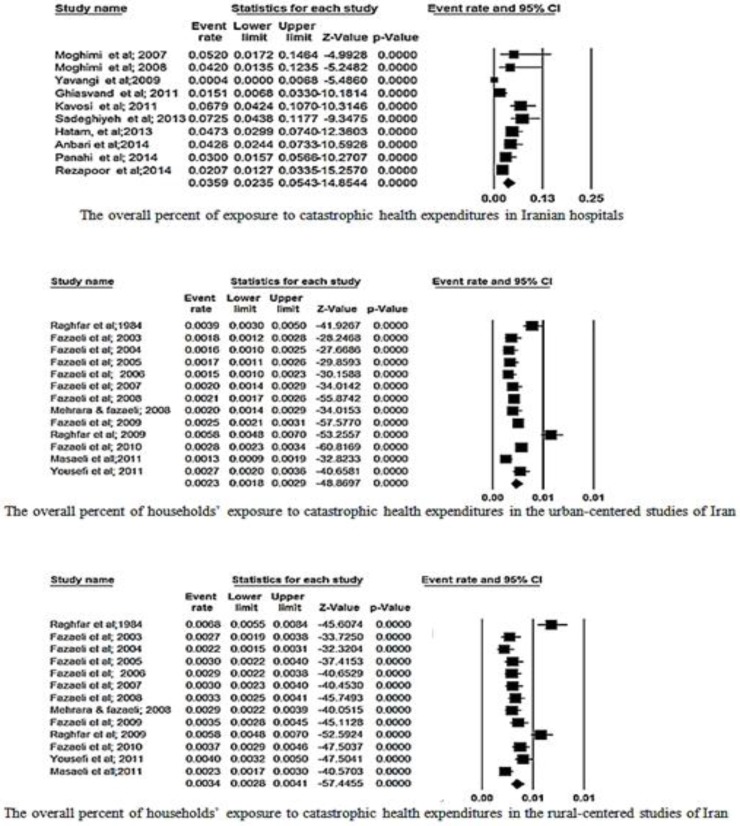
The overall percent of exposure to catastrophic health expenditures in Iranian hospitals, households in the urban-centered and rural-centered studies

Figures 2 and 3 address the analysis of data based on the data of the whole country, hospital-centered studies, urban and rural, respectively. Some studies calculated catastrophic expenditure in Iran based on household income and expenditure survey data carried out annually by the Statistical Center of Iran (SCI). Other studies have used the WHO survey or researcher constructed questionnaire to collect.

The overall percent of households’ exposure to catastrophic health expenditures in Iran based on random effect was 7.5% (95% CI, 6.2–9.1) (Fig. 2). About 95% CI for the present was drawn for each study in the horizontal line format (Q = 1628, df = 51, *P*<0.001 I^2^= 96.86). The overall percent of households exposed to catastrophic health expenditures in community-centered studies of Iran (rural and urban, without hospital) based on the random effect model was 5.4% (95% CI, 4.5–6.5, Q = 1208 df = 41, *P*<0.001 I^2^= 96.6).

According to Fig. 3, the overall percent of exposure to catastrophic health expenditures in Iranian hospitals based on the random effect was 35.9% (95% CI, 23.5–54.3, Q = 35.4, df = 9, *P*<0.001, I^2^=74.5). The overall percent of households exposure to catastrophic health expenditures in the urban-centered studies of Iran based on the random effect was 2.3% (95% CI, 1.8–2.9, Q=113.4, df =12, *P*<0.001, I^2^= 89.42). The overall percent of whom exposed to the catastrophic health expenditures in rural-centered studies of Iran based on the random effect was 3.4% (95% CI,2.8–4.1, Q= 86.08, df = 12, *P*<0.001, I^2^= 86.06).

**Fig. 4:**
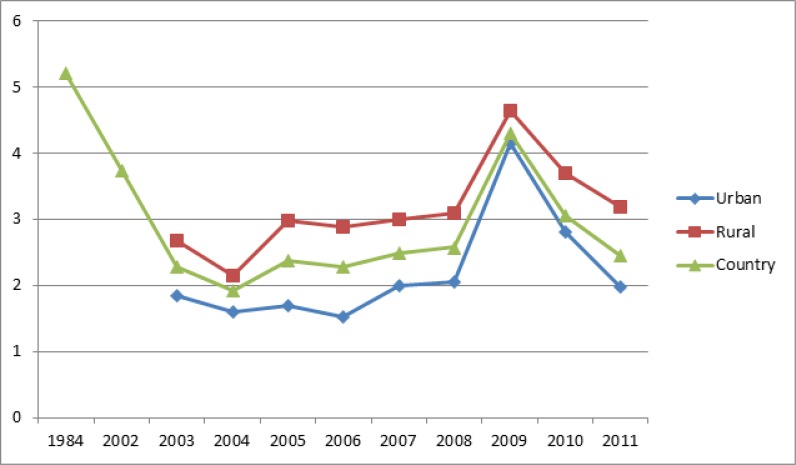
Catastrophic expenditures trend in Iran (1984–2011)

The overall percent of exposure to catastrophic health expenditures based on questionnaires on the random effect was 14.4% (95% CI, 10.9–19.1). 95% CI for the present was drawn for each study in the horizontal line format (Q = 220.3, df = 32, *P*<0.001, I^2^= 85.4). Moreover, the overall percent of exposure to catastrophic health expenditures using the income and expenditures surveys by SCI based on the random effect was 3.3% (95% CI, 2.6–4.3). 95% CI for the present was drawn for each study in the horizontal line format (Q =978.2, df = 19, *P*<0.001, I^2^=98.05). Finally, the trend of exposure to catastrophic expenditures in country-wide studies conducted based on data of the Statistical Center of Iran regarding household income and expenditure without the calculation of hospital-centered studies (Fig. 4).

## Discussion

Our review revealed that the incidence proportion of catastrophic expenditures is relatively high in Iran and it is necessary to adopt effective measures in this regard (64, 65).

Overall percentage of the exposure in published studies conducted between 1984 and 2015 was 7.5%. During the study years, the incidence proportion experienced fluctuations at some times. Catastrophic expenditures’ variation over time can be attributed to macro policies taken in the sectors of economy or health; however, other effective factors should be controlled to investigate this effect. For instance, family physician and rural insurance policies had not made much improvement in the trend of exposure to catastrophic expenditures in rural areas. Moreover, since the beginning of the implementation of this policy in 2005, the proportion of exposure to catastrophic expenditures had no significant decrease. The impacts of rural health insurance program were assessed and concluded that the program had not led to significant improvement in the status of vertical and horizontal equity among rural population (66). In the meanwhile, the targeted subsidy policy in 2010 shows a reduction in the trend of exposure to catastrophic expenditures throughout the country. However, the studies investigating the effect of targeted subsidies on health sector show that this policy had no impact on the equity in health financing (67), but a negative impact on health behavior of individuals, in particular among the poor, because households had allocated cash subsidies for their other needs rather than the health needs (68).

Levels of the exposure to catastrophic health expenditures in the urban and rural communities in the current study were estimated 2.3% and 3.4% respectively. Such difference might be attributed to the uneven distribution of healthcare facilities between urban and rural areas (69, 70). Rural insured people are covered by rural insurance funds and face problems in accessing health services and paying for them. Such problems occur if rural insured patients see a consultant physician out of the referral system ordered by a GP, which consequently private tariffs would be chargeable even if the patient chooses a public hospital. In addition, limited coverage by complementary health insurances among most rural population (71) makes them more vulnerable in paying for the health costs.

The proportion of exposure to catastrophic payments among urban community of Thailand, seven years after the implementation of the universal coverage policy, was 12.5% among the poor and 7.1% among other individuals. Catastrophic expenditures resulting from non-medical expenditures and indirect costs (lost time) were also calculated which were 30.4% for the poor and 18.3% for others (72). Low socio-economic status is as one of the factors affecting the exposure to catastrophic expenditures internationally. In most of low and middle-income countries, rich people usually allocate a larger share of household resources to their health, while the poor have to divert their resources into other basic needs and necessities such as food, house, and clothes (73). In Nigeria, the poorer households (the poor and villagers) experienced the highest burden of catastrophic expenditures (74). Considerable and unpredictable payments by households for health and a steady growth of medical bills which households have no budget for them may lead those households to face catastrophic expenditures (75).

In this review, suffering from chronic diseases was recognized as another important cause of exposure to catastrophic expenditures. In this regard, according to a systematic review, 84%–86% of households throughout the world, based on various thresholds (10%–40%) have faced catastrophic expenditures due to non-communicable diseases. Non-communicable diseases have shown a vast and growing global impact on households’ poverty in all countries and among all income levels. However, the actual impact of chronic diseases on households’ poverty is usually underestimated, because information about those living in marginalized areas and vulnerable ones who do not seek healthcare are not recorded (76).

The status of coverage by health insurance, as well as the coverage level of services by health insurance, is other causes of the exposure to catastrophic expenditures. Low coverage of services and high levels of co-payment mean that households experience higher risk of catastrophic expenditures and economic hardship (77).

Most studies conducted in Iran mentioned inpatient services as factors that increase the possibility of the exposure to catastrophic expenditures. Costs of hospitalization, in particular for long-term stay and frequent hospitalization in Intensive Care Units (ICU), which embody high bed-day costs, significantly increase payable bills for patients; and increase the probability of the exposure to catastrophic expenditures. In the meanwhile, the higher inpatient services charges in private hospitals put many households at the risk of exposure to catastrophic expenditures, particularly when long waiting list at public sector forces patients to go to a private hospital. In our review, hospital-centered studies were conducted in internal medicine, surgical and oncology wards, intensive care units, dialysis units as well as major departments in general hospitals.

The majority of the Iranian studies have introduced the hospitalization in private hospital as a factor affecting the incidence of catastrophic expenditures. However, in Nigeria, the incidence of catastrophic expenditures for outpatient services was higher than those for inpatient ones (74).

In our review, the mean of exposure to catastrophic expenditures resulted hospitalization was 35.9%. Although a study conducted in India revealed that 84% of patients experienced catastrophic expenditures resulting from the treatment of acute coronary syndrome which is the main death cause in coronary disease (78).

Our review could be stronger if more hospital-based, service-based and disease-based studies were available. Hence, such studies are recommended to be conducted.

## Conclusion

Since our review indicated the utilization of inpatient services as an important factor in the incidence of catastrophic expenditures and given that limited studies were conducted in the field of catastrophic expenditures of hospital services in Iran, further studies are suggested to be conducted in inpatient services and different provinces of Iran. In addition, further studies about the incidence of catastrophic expenditures in para-clinic services, certain expensive medicines, and outpatient services are recommended.

## Ethical considerations

Ethical issues (Including plagiarism, informed consent, misconduct, data fabrication and/or falsification, double publication and/or submission, redundancy, etc.) have been completely observed by the authors.

## References

[1] Mooney G (1993). Equity in the finance and delivery of health care, an international perspective. J Epidemiol Community Health, 47(4): 338–39.

[2] World Health Organization (2000). The World health report: 2000: Health systems: improving performance.

[3] Rezapour A Ghaderi H Azar FE Larijani B Gohari MR (2013). Effects of Health Out-of-Pocket Payment on HouseHolds in Iran; Catastrophic and Impoverishment: Population Based Study in Tehran (2012). Life Sci J, 10(3): 1457–69.

[4] Jan S Lee SW Sawhney JP (2016). Catastrophic health expenditure on acute coronary events in Asia: a prospective study. Bull World Health Organ, 94(3): 193–200. 26966330 10.2471/BLT.15.158303 PMC4773930

[5] Cotlear D Nagpal S Smith O Tandon A Cortez R (2015). Going Universal: How 24 developing countries are implementing Universal Health Coverage Reforms. Washington DC, World Bank.

[6] Xu K Ravndal F Evans DB Carrin G (2009). Assessing the reliability of household expenditure data: results of the World Health Survey. Health Policy, 91(3): 297–305. 19217184 10.1016/j.healthpol.2009.01.002

[7] Onoka CA Onwujekwe OE Hanson K Uzochukwu BS (2011). Examining catastrophic health expenditures at variable thresholds using household consumption expenditure diaries. Trop Med Int Health, 16(10): 1334–41. 21752164 10.1111/j.1365-3156.2011.02836.x

[8] O’Donnell O Van Doorslaer E Wagstaff A Lindelow M (2008). Catastrophic payments for health care. Analyzing Health Equity Using Household Survey Data. Washington, DC: The World Bank.

[9] World Health Organization (2012). Spending on health: A global overview. Fact sheet. Geneva, 319.

[10] Buigut S Ettarh R Amendah DD (2015). Catastrophic health expenditure and its determinants in Kenya slum communities. Int J Equity Health, 14;14:46. 25971679 10.1186/s12939-015-0168-9 PMC4438568

[11] Kavosi Z Rashidian A Pourreza A (2012). Inequality in household catastrophic health care expenditure in a low-income society of Iran. Health Policy Plan, 27(7): 613–23. 22279081 10.1093/heapol/czs001

[12] World Health Organization (2015). Tracking universal health coverage: first global monitoring report. ed. World Health Organization.

[13] Ghosh S (2010). Catastrophic payments and Impoverishment due to Out-of-Pocket health spending: The effects of recent health sector reforms in India. Econ Polit Wkly, 46(47): 63–70.

[14] World Health Organization (2014). WHO Global Health Expenditure Atlas. ed. World Health Organization, Switzerland, 224–224.

[15] Murray CJ Evans D (2006). Health systems performance assessment. Office of Health Economics.

[16] Xu K Evans DB Carrin G Aguilar-Rivera AM Musgrove P Evans T (2007). Protecting households from catastrophic health spending. Health Aff (Millwood), 26(4): 972–83. 17630440 10.1377/hlthaff.26.4.972

[17] Xu K Evans DB Kawabata K Zeramdini R Klavus J Murray CJ (2003). Household catastrophic health expenditure: a multicountry analysis. Lancet, 362(9378): 111–7. 12867110 10.1016/S0140-6736(03)13861-5

[18] Barros AJ Bertoldi AD (2008). Out-of-pocket health expenditure in a population covered by the Family Health Program in Brazil. Int J Epidemiol, 37(4): 758–65. 18411201 10.1093/ije/dyn063

[19] Su TT Kouyaté B Flessa S (2006). Catastrophic household expenditure for health care in a low-income society: a study from Nouna District, Burkina Faso. Bull World Health Organ, 84(1): 21–7. 16501711 10.2471/blt.05.023739 PMC2626518

[20] Ghiasvand H Gorji HA Maleki M Hadian M (2015). Catastrophic Health Expenditure Among Iranian Rural and Urban Households, 2013–2014. Iran Red Crescent Med J, 17(9): e30974. 26473081 10.5812/ircmj.30974 PMC4601211

[21] Masaeli A Sadeghih H Ghanbari A (2011). High Health Costs, Financial Catastrophic and Impoverishment Expenditures: Concepts for Policy Formation. Health Inf Manage, 12(2):254–61

[22] Sadeghiyehahari S Amani F Salehi M (2013). Investigation of Exposure to Catastrophic Expenditures in Patients with End Stage Renal Disease Refering to Ardebil Dialysis Centers Ardabil University of Medical Science. Master of Sciences Thesis. Ardabil University of Medical Science.

[23] Ameri H Vafayi H Alizadeh H Ghiyasi A Razavi NS Khalfi A (2013). Estimates of Catastrophic Health Care Expenditures on Families Supported by Torbat Heydariyeh University of Medical Sciences in 1391. JTHUMS, 1(2): 46–54.

[24] Tofighi S Asefzadeh S Mamikhani J Sadeghifar J (2014). The Impact of Rural Health Insurance on Reduction of Catastrophic Health Expenditure (CHE). JAEBS, 4(5): 154–60.

[25] Asefzadeh S Alijanzadeh M Gholamalipoor S Farzaneh A (2013). Households Encountering with Catastrophic Health Expenditures in Qazvin, Iran. Health Inf Manage, 10(1): 1–8.

[26] Wells G Shea B O’connell D Peterson J Welch V Losos M Tugwell P (2000). The Newcastle-Ottawa Scale (NOS) for assessing the quality of nonrandomised studies in meta-analyses. Ottawa Hospital Research Institute.

[27] Higgins JP Thompson SG Deeks JJ Altman DG (2003). Measuring inconsistency in meta-analyses. BMJ, 327 (7414): 557–560. 12958120 10.1136/bmj.327.7414.557 PMC192859

[28] Abolhallaje M Hasani SA Bastani P Ramezanian M Kazemian M (2013). Determinants of catastrophic health expenditure in Iran. Iran J Public Health, 155–160. 23865034 PMC3712590

[29] Ameri H Jafari A Panahi M (2013). Determining the Rate of Catastrophic Health Expenditure and Its Influential Factors on Families in Yazd Province. J Health Adm, 16(52): 51–60.

[30] Anbari Z Mohammadbeigi A Mohammadsalehi N Ebrazeh A (2014). Health expenditure and catastrophic costs for inpatient- and out-patient care in Iran. Int J Prev Med, 5 (8): 1023–1028. 25489451 PMC4258666

[31] AsadiLari M Vaez-Mahdavi MR (2008). An overview on Urban-HEART Tehran experience. World Health Organization.

[32] Bagherifaradonbeh S Arab M Roodbari M Rezapoor A Bagherifaradonbeh Hazar FE (2016). Catastrophic and Impoverishing Health Expenditure in Tehran Urban Population. J Health Adm, 19 (63): 55–67.

[33] Daneshkohan A Karami M Najafi F Karami Matin B (2011). Household catastrophic health expenditure. Iran J Public Health, 40(1): 94–99. 23113061 PMC3481728

[34] Fazaeli AA Ghaderi H Fazaeli AA Lotfi F Salehi M Mehrara M (2015). Main Determinants of Catastrophic Health Expenditures: A Bayesian Logit Approach on Iranian Household Survey Data (2010). Glob J Health Sci, 7(4): 335–40. 10.5539/gjhs.v7n4p335 PMC4802182 25946936

[35] Fazaeli AA Seyedin H Moghaddam AV Delavari A Salimzadeh H Varmazyar H Fazaeli AA (2015). Fairness of Financial Contribution in Iranian Health System: Trend Analysis of National Household Income and Expenditure, 2003–2010. Glob J Health Sci, 7(5): 260–5 10.5539/gjhs.v7n5p260 PMC4803859 26156920

[36] Ghafoori MH Ebadifard Azar F Arab M Mahmoodi M Yusef Zadeh N Rezapour A (2014). The Distribution of Health Expenditures in Tehran’s Districts. JCHR, 3(2): 132–44.

[37] Ghiasi A Baghi A Rezapour A Alipour V Ahadinezhad B Mahmoudi M Veysinasab F (2016). Health insurance, medicine expenses and catastrophic health expenditures. J Health Adm, 18(62): 64–73.

[38] Ghiasvand H Hadian M Maleki M Shabaninejad H (2010). Determinants of Catastrophic Medical Payments in Hospitals Affiliated to Iran University of Medical Sciences; 2009. Hakim Health Sys Res, 13(3): 145–54.

[39] Ghiasvand H Sha’baninejad H Arab M Rashidian A (2014). Hospitalization and catastrophic medical payment: Evidence from hospitals located in Tehran. Arch Iran Med, 17(7): 507–13. 24979565

[40] Ghoddoosinejad J Jannati A Gholipour K Baghestan EB (2014). Households encountering with catastrophic health expenditures in Ferdows, Iran. J Egypt Public Health Assoc, 89(2): 81–4. 25162739 10.1097/01.EPX.0000451789.21421.61

[41] Hajizadeh M Nghiem HS (2011). Out-of-pocket expenditures for hospital care in Iran: Who is at risk of incurring catastrophic payments? Int J Health Care Finance Econ, 11(4): 267–85. 21915727 10.1007/s10754-011-9099-1

[42] Hanjani HM Fazaeli A (2005). Estimation of Fair Financial Contribution in Health System of Iran. Social walfare, 5(19): 279–300.

[43] Hatam N Orejlu PH Jafari A Kavosi Z (2015). Catastrophic Healthcare Expenditures of Hospitalized Patients in the Hospitals of Shiraz in 2013. Shiraz E-Med J, 16(5): e22231

[44] Juyani Y Hamedi D Jebeli SSH Qasham M (2016). Multiple Sclerosis and Catastrophic Health Expenditure in Iran. Glob J Health Sci, 8(9): 53778. 27157166 10.5539/gjhs.v8n9p194 PMC5064064

[45] Karami M Najafi F Karami Matin B (2009). Catastrophic health expenditures in Kermanshah, West of Iran: Magnitude and distribution. J Res Health Sci, 9(2): 36–40. 23344170

[46] Kavosi Z Delavari H Keshtkaran A Setoudehzadeh F (2014). Catastrophic health expenditures and coping strategies in households with cancer patients in Shiraz Namazi Hospital. Middle East J Cancer, 5(1): 13–22.

[47] Kavosi Z Keshtkaran A Hayati R Ravangard R Khammarnia M (2014). Household financial contribution to the health System in Shiraz, Iran in 2012. Int J Health Policy Manag, 3(5): 243–249. 25337598 10.15171/ijhpm.2014.87 PMC4204743

[48] Mehrara M Fazaeli A (2010). Health finance equity in Iran: an analysis of household survey data (1382–1386). J Health Adm, 13(40):51–62.

[49] Moghimi M Zanjani SV Zamiri RE Feizi A Rostamkhani MH Ghahramani R (2008). Performance of Government Rule in Supprting and Decreasing Catastrophic Expenditures of Cancer Patients in Zanjan Province in 2007–2008. Health System, 1(2):41–46.

[50] Nekoei Moghadam M Akbari-Javar M Amiresmaili M Baneshi M Ganjavai S (2013). Households Exposure to Catastrophic Health Expenditures and the Affecting Factors in Kerman Province, Iran. J Manage Med Inform Sch, 101–90.

[51] Moghadam MN Baneshi M Javar MA Amiresmaili M Ganjavi S (2012). Iranian household financial protection against catastrophic health care expenditures. Iran J Public Health, 41(9):62–70. 23193508 PMC3494217

[52] Panahi H Janati A Narimani M (2014). Catastrophic Expenditure for Hospitalized Patients in Tabriz, Iran. Payesh, 13(6): 655–663.

[53] Piroozi B Moradi G Nouri B Mohamadi Bolbanabad A Safari H (2016). Catastrophic health expenditure after the implementation of health sector evolution plan: a case study in the west of Iran. Int J Health Policy Manag, 5(7):417–423. 27694669 10.15171/ijhpm.2016.31 PMC4930347

[54] Raghfar H AtrkarRoshan S Atefi M (2013). Measurement of the Fair Financial Contribution Index and Catastrophic Expenditures in Different Regions of Iran, 1984–2010. Hakim Health Sys Res, 16(3): 182–191.

[55] Razavi SM (2011). Impact of Structural Adjustment Programs on Health Care Financing in Iran. Brandeis University, The Heller School for Social Policy and Management.

[56] Rezapour A Arabloo J Tofighi S Alipour V Sepandy M Mokhtari P Ghanbary A (2016). Determining Equity in Household’s Health Care Payments in Hamedan Province, Iran. Arch Iran Med, 19(7): 480–87. 27362241

[57] Rezapour A Azar AE Asadi S Faradonbeh SB Toofan F (2016). Estimating the Odd-Ratio of Factors Affecting Households’ Exposure to Catastrophic and Impoverishing Health Expenditures. J Mil Med, 18(1): 355–61.

[58] Sabermahani A Noranimotlagh S Vaezmahdavi M Hadian M Asadilari M (2014). Catastrophic Health Expenditures and its Determinants among Households in Tehran in 2011, Urban HEART-2 study. RJMS, 21(126): 15–26.

[59] Soofi M Rashidian A Aabolhasani F Sari AA Bazyar M (2013). Measuring the Exposure of Households to Catastrophic Healthcare Expenditures in Iran in 2001: the World Health Organization and the World Bank’s Approach. Hospital, 12(2): 39–50.

[60] Tofighi S Zaboli R Mahdavi MRV (2015). The Healthcare Costs in the Aging Based on Data from the Urban Health Equity Assessment and Response Tool Project in Tehran, Iran (UHEART-2). IJMR, 2(1): 201–207.

[61] Yavangi M Sohrabi MR Riazi S (2013). Out of pocket payment for obstetrical complications: a cost analysis study in iran. Int J Prev Med, 4(11): 1296–1303. 24404365 PMC3883255

[62] Yousefi M Assari Arani A Sahabi B Kazemnejad A Fazaeli S (2015). The Financial Contribution Of Households Using By Health Services. Payavard Salamat, 8(6): 517–27

[63] Zare H Trujillo AJ Driessen J Ghasemi M Gallego G (2014). Health inequalities and development plans in Iran; an analysis of the past three decades (1984–2010). Int J Equity Health, 13(1): 42 24885492 10.1186/1475-9276-13-42 PMC4046006

[64] Aryankhesal A Etemadi M Agharahimi Z Rostami E Mohseni M (2016). Analysis of social functions in Iran’s public hospitals: pattern of offering discounts to poor patients. Int J Hum Rights Healthc, 9(4): 242–53.

[65] Mahdavi M Parsaeian M Jaafaripooyan E Ghaffari E (2017). Recent iranian health system reform: an operational perspective to improve health services quality. Int J Health Policy Manag, 7(1): 70–74. 29325404 10.15171/ijhpm.2017.89 PMC5745869

[66] Khosravi M Kazemian M (2013). The Evaluation of rural health insurance plan based on horizontal and vertical equities situation. IJHIE, 1(1):1–6.

[67] Zandian H Olyaeemanesh A Takian A Hosseini M (2016). Contribution of Targeted Subsidies Law to the Equity in Healthcare Financing in Iran: Exploring the Challenges of Policy Process. Electronic Physician, 8(2): 1892–1903. 27053996 10.19082/1892 PMC4821302

[68] Doshmangir L Doshmangir P Abolhassani N Moshiri E Jafari M (2015). Effects of Targeted Subsidies Policy on Health Behavior in Iranian Households: A Qualitative Study. Iran J Public Health, 44(4):570–9. 26056676 PMC4441970

[69] Abolhallaje M Mousavi SM Anjomshoa M (2014). Assessing health inequalities in Iran: a focus on the distribution of health care facilities. Glob J Health Sci, 6(4):285–91. 24999132 10.5539/gjhs.v6n4p285 PMC4825387

[70] Sabermahani A Barouni M Seyedin H Aryankhesal A (2013). Provincial human development index, a guide for efficiency level analysis: the case of iran. Iran J Public Health, 42(2):149–57 23515434 PMC3595646

[71] Lankarani KB Ghahramani S Zakeri M Joulaei H (2015). Lessons learned from national health accounts in Iran: highlighted evidence for policymakers. Shiraz E Med J, 16(4): 1–3

[72] Weraphong J Pannarunothai S Luxananun T Junsri N Deesawatsripetch S (2013). Catastrophic health expenditure in an urban city: seven years after universal coverage policy in Thailand. Southeast Asian J Trop Med Public Health, 44(1):124–36. 23682447

[73] Van Doorslaer E O’Donnell O Rannan-Eliya RP (2007). Catastrophic payments for health care in Asia. Health Econ, 16(11):1159–84. 17311356 10.1002/hec.1209

[74] Onwujekwe O Hanson K Uzochukwu B (2012). Examining inequities in incidence of catastrophic health expenditures on different healthcare services and health facilities in Nigeria. PLoS One, 7(7): e40811. 22815828 10.1371/journal.pone.0040811 PMC3397929

[75] Essue BM Kimman M Svenstrup N Kjoege KL Laba TL Hackett ML Jan S (2015). The effectiveness of interventions to reduce the household economic burden of illness and injury: A systematic review. Bull World Health Organ, 93(2):102–12B. 25883403 10.2471/BLT.14.139287 PMC4339963

[76] Jaspers L Colpani V Chaker L (2014). The global impact of non-communicable diseases on households and impoverishment: a systematic review. Eur J Epidemiol, 30(3):163–88. 25527371 10.1007/s10654-014-9983-3

[77] Wagstaff A Lindelow M (2008). Can insurance increase financial risk?: The curious case of health insurance in China. J Health Econ, 27(4):990–1005. 18342963 10.1016/j.jhealeco.2008.02.002

[78] Daivadanam M Thankappan K Sarma P Harikrishnan S (2012). Catastrophic health expenditure & coping strategies associated with acute coronary syndrome in Kerala, India. Indian J Med Res, 136(4):585–92. 23168698 PMC3516025

